# Supplementary material for: Pyruvate Dehydrogenase Kinase 1 inhibition mediated oxidative phosphorylation enhancement in cartilage promotes osteoarthritis progression
Source: BMC Musculoskelet Disord. 2023 Jul 20;24:597. doi: 10.1186/s12891-023-06585-6 (PMC10357736; doi:10.1186/s12891-023-06585-6)
Supplement: Supplementary file 1 — Additional file 1. [file 12891_2023_6585_MOESM1_ESM.zip › MF.Gsea.1653627316242/GOMF_MRNA_BINDING.html]

Details for gene set GOMF\_MRNA\_BINDING[GSEA]

|  || Dataset | 55457\_collapsed\_to\_symbols.cls.cls #OA\_versus\_normal.cls.cls #OA\_versus\_normal\_repos |
| Phenotype | cls.cls#OA\_versus\_normal\_repos |
| Upregulated in class | normal |
| GeneSet | GOMF\_MRNA\_BINDING |
| Enrichment Score (ES) | -0.44679862 |
| Normalized Enrichment Score (NES) | -2.226474 |
| Nominal p-value | 0.0 |
| FDR q-value | 0.001095823 |
| FWER p-Value | 0.001 |
Table: GSEA Results Summary

  

Fig 1: Enrichment plot: GOMF\_MRNA\_BINDING      
 Profile of the Running ES Score & Positions of GeneSet Members on the Rank Ordered List

  

| SYMBOL | TITLE | RANK IN GENE LIST | RANK METRIC SCORE | RUNNING ES | CORE ENRICHMENT || 1 | PCBP1 | poly(rC) binding protein 1 [Source:HGNC Symbol;Acc:HGNC:8647] | 197 | 0.823 | -0.0064 | No |
| 2 | PPIE | peptidylprolyl isomerase E [Source:HGNC Symbol;Acc:HGNC:9258] | 334 | 0.733 | -0.0089 | No |
| 3 | TSN | translin [Source:HGNC Symbol;Acc:HGNC:12379] | 372 | 0.717 | -0.0038 | No |
| 4 | SLC4A1AP | solute carrier family 4 member 1 adaptor protein [Source:HGNC Symbol;Acc:HGNC:13813] | 664 | 0.602 | -0.0201 | No |
| 5 | RBM4B | RNA binding motif protein 4B [Source:HGNC Symbol;Acc:HGNC:28842] | 681 | 0.598 | -0.0147 | No |
| 6 | CRYZ | crystallin zeta [Source:HGNC Symbol;Acc:HGNC:2419] | 788 | 0.572 | -0.0167 | No |
| 7 | PPP1R8 | protein phosphatase 1 regulatory subunit 8 [Source:HGNC Symbol;Acc:HGNC:9296] | 885 | 0.547 | -0.0182 | No |
| 8 | TACO1 | translational activator of cytochrome c oxidase I [Source:HGNC Symbol;Acc:HGNC:24316] | 923 | 0.536 | -0.0151 | No |
| 9 | TDRD7 | tudor domain containing 7 [Source:HGNC Symbol;Acc:HGNC:30831] | 1020 | 0.513 | -0.0170 | No |
| 10 | MRPS11 | mitochondrial ribosomal protein S11 [Source:HGNC Symbol;Acc:HGNC:14050] | 1305 | 0.457 | -0.0343 | No |
| 11 | PARK7 | Parkinsonism associated deglycase [Source:HGNC Symbol;Acc:HGNC:16369] | 1406 | 0.442 | -0.0373 | No |
| 12 | CARHSP1 | calcium regulated heat stable protein 1 [Source:HGNC Symbol;Acc:HGNC:17150] | 1498 | 0.429 | -0.0397 | No |
| 13 | SHMT2 | serine hydroxymethyltransferase 2 [Source:HGNC Symbol;Acc:HGNC:10852] | 1518 | 0.426 | -0.0364 | No |
| 14 | MRPS7 | mitochondrial ribosomal protein S7 [Source:HGNC Symbol;Acc:HGNC:14499] | 1650 | 0.406 | -0.0422 | No |
| 15 | MRPL13 | mitochondrial ribosomal protein L13 [Source:HGNC Symbol;Acc:HGNC:14278] | 1655 | 0.406 | -0.0380 | No |
| 16 | DDX6 | DEAD-box helicase 6 [Source:HGNC Symbol;Acc:HGNC:2747] | 1687 | 0.401 | -0.0360 | No |
| 17 | NUDT21 | nudix hydrolase 21 [Source:HGNC Symbol;Acc:HGNC:13870] | 1734 | 0.393 | -0.0352 | No |
| 18 | MBD2 | methyl-CpG binding domain protein 2 [Source:HGNC Symbol;Acc:HGNC:6917] | 1958 | 0.366 | -0.0488 | No |
| 19 | DXO | decapping exoribonuclease [Source:HGNC Symbol;Acc:HGNC:2992] | 1960 | 0.366 | -0.0447 | No |
| 20 | RBM4 | RNA binding motif protein 4 [Source:HGNC Symbol;Acc:HGNC:9901] | 1970 | 0.364 | -0.0414 | No |
| 21 | NHP2 | NHP2 ribonucleoprotein [Source:HGNC Symbol;Acc:HGNC:14377] | 2026 | 0.358 | -0.0417 | No |
| 22 | ESRP1 | epithelial splicing regulatory protein 1 [Source:HGNC Symbol;Acc:HGNC:25966] | 2064 | 0.355 | -0.0407 | No |
| 23 | SCAF8 | SR-related CTD associated factor 8 [Source:HGNC Symbol;Acc:HGNC:20959] | 2207 | 0.337 | -0.0481 | No |
| 24 | IREB2 | iron responsive element binding protein 2 [Source:HGNC Symbol;Acc:HGNC:6115] | 2215 | 0.337 | -0.0449 | No |
| 25 | ESRP2 | epithelial splicing regulatory protein 2 [Source:HGNC Symbol;Acc:HGNC:26152] | 2435 | 0.310 | -0.0588 | No |
| 26 | MYEF2 | myelin expression factor 2 [Source:HGNC Symbol;Acc:HGNC:17940] | 2465 | 0.307 | -0.0576 | No |
| 27 | SRBD1 | S1 RNA binding domain 1 [Source:HGNC Symbol;Acc:HGNC:25521] | 2571 | 0.294 | -0.0627 | No |
| 28 | ANGEL2 | angel homolog 2 [Source:HGNC Symbol;Acc:HGNC:30534] | 2608 | 0.290 | -0.0623 | No |
| 29 | NYNRIN | NYN domain and retroviral integrase containing [Source:HGNC Symbol;Acc:HGNC:20165] | 2691 | 0.280 | -0.0656 | No |
| 30 | APOBEC1 | apolipoprotein B mRNA editing enzyme catalytic subunit 1 [Source:HGNC Symbol;Acc:HGNC:604] | 2695 | 0.280 | -0.0627 | No |
| 31 | CSTF2T | cleavage stimulation factor subunit 2 tau variant [Source:HGNC Symbol;Acc:HGNC:17086] | 2749 | 0.275 | -0.0638 | No |
| 32 | IGF2BP3 | insulin like growth factor 2 mRNA binding protein 3 [Source:HGNC Symbol;Acc:HGNC:28868] | 2770 | 0.273 | -0.0624 | No |
| 33 | POLDIP3 | DNA polymerase delta interacting protein 3 [Source:HGNC Symbol;Acc:HGNC:23782] | 2869 | 0.264 | -0.0672 | No |
| 34 | SLPI | secretory leukocyte peptidase inhibitor [Source:HGNC Symbol;Acc:HGNC:11092] | 2903 | 0.260 | -0.0669 | No |
| 35 | RAVER2 | "ribonucleoprotein, PTB binding 2 [Source:HGNC Symbol;Acc:HGNC:25577]" | 2956 | 0.254 | -0.0681 | No |
| 36 | SHFL | shiftless antiviral inhibitor of ribosomal frameshifting [Source:HGNC Symbol;Acc:HGNC:25649] | 3006 | 0.249 | -0.0692 | No |
| 37 | FXR1 | FMR1 autosomal homolog 1 [Source:HGNC Symbol;Acc:HGNC:4023] | 3012 | 0.249 | -0.0668 | No |
| 38 | C1QBP | complement C1q binding protein [Source:HGNC Symbol;Acc:HGNC:1243] | 3083 | 0.241 | -0.0697 | No |
| 39 | PARN | poly(A)-specific ribonuclease [Source:HGNC Symbol;Acc:HGNC:8609] | 3100 | 0.240 | -0.0683 | No |
| 40 | PEG10 | paternally expressed 10 [Source:HGNC Symbol;Acc:HGNC:14005] | 3118 | 0.238 | -0.0669 | No |
| 41 | LIN28A | lin-28 homolog A [Source:HGNC Symbol;Acc:HGNC:15986] | 3140 | 0.236 | -0.0660 | No |
| 42 | CSTF3 | cleavage stimulation factor subunit 3 [Source:HGNC Symbol;Acc:HGNC:2485] | 3426 | 0.209 | -0.0862 | No |
| 43 | CIRBP | cold inducible RNA binding protein [Source:HGNC Symbol;Acc:HGNC:1982] | 3441 | 0.207 | -0.0850 | No |
| 44 | KHNYN | KH and NYN domain containing [Source:HGNC Symbol;Acc:HGNC:20166] | 3457 | 0.206 | -0.0838 | No |
| 45 | RIDA | reactive intermediate imine deaminase A homolog [Source:HGNC Symbol;Acc:HGNC:16897] | 3488 | 0.203 | -0.0839 | No |
| 46 | RBFOX2 | RNA binding fox-1 homolog 2 [Source:HGNC Symbol;Acc:HGNC:9906] | 3523 | 0.200 | -0.0844 | No |
| 47 | SNRPC | small nuclear ribonucleoprotein polypeptide C [Source:HGNC Symbol;Acc:HGNC:11157] | 3649 | 0.190 | -0.0921 | No |
| 48 | SLBP | stem-loop binding protein [Source:HGNC Symbol;Acc:HGNC:10904] | 3703 | 0.185 | -0.0943 | No |
| 49 | KHDRBS3 | "KH RNA binding domain containing, signal transduction associated 3 [Source:HGNC Symbol;Acc:HGNC:18117]" | 3714 | 0.184 | -0.0930 | No |
| 50 | KHDRBS1 | "KH RNA binding domain containing, signal transduction associated 1 [Source:HGNC Symbol;Acc:HGNC:18116]" | 3748 | 0.182 | -0.0936 | No |
| 51 | PKM | pyruvate kinase M1/2 [Source:HGNC Symbol;Acc:HGNC:9021] | 3963 | 0.159 | -0.1087 | No |
| 52 | EIF3D | eukaryotic translation initiation factor 3 subunit D [Source:HGNC Symbol;Acc:HGNC:3278] | 4032 | 0.153 | -0.1124 | No |
| 53 | MYH10 | myosin heavy chain 10 [Source:HGNC Symbol;Acc:HGNC:7568] | 4169 | 0.141 | -0.1216 | No |
| 54 | YTHDF1 | YTH N6-methyladenosine RNA binding protein 1 [Source:HGNC Symbol;Acc:HGNC:15867] | 4320 | 0.127 | -0.1320 | No |
| 55 | TYMS | thymidylate synthetase [Source:HGNC Symbol;Acc:HGNC:12441] | 4328 | 0.126 | -0.1312 | No |
| 56 | A1CF | APOBEC1 complementation factor [Source:HGNC Symbol;Acc:HGNC:24086] | 4368 | 0.123 | -0.1329 | No |
| 57 | SNRNP35 | small nuclear ribonucleoprotein U11/U12 subunit 35 [Source:HGNC Symbol;Acc:HGNC:30852] | 4547 | 0.109 | -0.1457 | No |
| 58 | KHDRBS2 | "KH RNA binding domain containing, signal transduction associated 2 [Source:HGNC Symbol;Acc:HGNC:18114]" | 4550 | 0.109 | -0.1447 | No |
| 59 | PCBP4 | poly(rC) binding protein 4 [Source:HGNC Symbol;Acc:HGNC:8652] | 4577 | 0.107 | -0.1455 | No |
| 60 | PTBP1 | polypyrimidine tract binding protein 1 [Source:HGNC Symbol;Acc:HGNC:9583] | 4650 | 0.100 | -0.1501 | No |
| 61 | GRSF1 | G-rich RNA sequence binding factor 1 [Source:HGNC Symbol;Acc:HGNC:4610] | 4660 | 0.099 | -0.1497 | No |
| 62 | EIF4G3 | eukaryotic translation initiation factor 4 gamma 3 [Source:HGNC Symbol;Acc:HGNC:3298] | 4675 | 0.098 | -0.1497 | No |
| 63 | LARP6 | "La ribonucleoprotein 6, translational regulator [Source:HGNC Symbol;Acc:HGNC:24012]" | 4798 | 0.087 | -0.1584 | No |
| 64 | HNRNPD | heterogeneous nuclear ribonucleoprotein D [Source:HGNC Symbol;Acc:HGNC:5036] | 4830 | 0.085 | -0.1599 | No |
| 65 | EIF2S2 | eukaryotic translation initiation factor 2 subunit beta [Source:HGNC Symbol;Acc:HGNC:3266] | 4838 | 0.084 | -0.1595 | No |
| 66 | DAZ1 | deleted in azoospermia 1 [Source:HGNC Symbol;Acc:HGNC:2682] | 4920 | 0.078 | -0.1650 | No |
| 67 | G3BP2 | G3BP stress granule assembly factor 2 [Source:HGNC Symbol;Acc:HGNC:30291] | 5001 | 0.073 | -0.1705 | No |
| 68 | CDC40 | cell division cycle 40 [Source:HGNC Symbol;Acc:HGNC:17350] | 5228 | 0.054 | -0.1878 | No |
| 69 | EIF4ENIF1 | eukaryotic translation initiation factor 4E nuclear import factor 1 [Source:HGNC Symbol;Acc:HGNC:16687] | 5330 | 0.045 | -0.1953 | No |
| 70 | RPS26 | ribosomal protein S26 [Source:HGNC Symbol;Acc:HGNC:10414] | 5368 | 0.043 | -0.1977 | No |
| 71 | STRAP | serine/threonine kinase receptor associated protein [Source:HGNC Symbol;Acc:HGNC:30796] | 5375 | 0.042 | -0.1977 | No |
| 72 | HNRNPA0 | heterogeneous nuclear ribonucleoprotein A0 [Source:HGNC Symbol;Acc:HGNC:5030] | 5485 | 0.033 | -0.2060 | No |
| 73 | TP53 | tumor protein p53 [Source:HGNC Symbol;Acc:HGNC:11998] | 5511 | 0.032 | -0.2076 | No |
| 74 | NXF3 | nuclear RNA export factor 3 [Source:HGNC Symbol;Acc:HGNC:8073] | 5648 | 0.020 | -0.2181 | No |
| 75 | EXOSC4 | exosome component 4 [Source:HGNC Symbol;Acc:HGNC:18189] | 5693 | 0.016 | -0.2214 | No |
| 76 | DHFR | dihydrofolate reductase [Source:HGNC Symbol;Acc:HGNC:2861] | 5805 | 0.008 | -0.2301 | No |
| 77 | HDLBP | high density lipoprotein binding protein [Source:HGNC Symbol;Acc:HGNC:4857] | 5879 | 0.004 | -0.2358 | No |
| 78 | AUH | AU RNA binding methylglutaconyl-CoA hydratase [Source:HGNC Symbol;Acc:HGNC:890] | 5911 | 0.001 | -0.2383 | No |
| 79 | TPR | "translocated promoter region, nuclear basket protein [Source:HGNC Symbol;Acc:HGNC:12017]" | 5919 | 0.001 | -0.2388 | No |
| 80 | RBMXL2 | RBMX like 2 [Source:HGNC Symbol;Acc:HGNC:17886] | 6034 | -0.007 | -0.2477 | No |
| 81 | N4BP1 | NEDD4 binding protein 1 [Source:HGNC Symbol;Acc:HGNC:29850] | 6040 | -0.007 | -0.2480 | No |
| 82 | FUBP3 | far upstream element binding protein 3 [Source:HGNC Symbol;Acc:HGNC:4005] | 6115 | -0.014 | -0.2537 | No |
| 83 | PIWIL1 | piwi like RNA-mediated gene silencing 1 [Source:HGNC Symbol;Acc:HGNC:9007] | 6144 | -0.016 | -0.2558 | No |
| 84 | RBFOX1 | RNA binding fox-1 homolog 1 [Source:HGNC Symbol;Acc:HGNC:18222] | 6152 | -0.016 | -0.2561 | No |
| 85 | DAZL | deleted in azoospermia like [Source:HGNC Symbol;Acc:HGNC:2685] | 6221 | -0.022 | -0.2613 | No |
| 86 | TEX13A | testis expressed 13A [Source:HGNC Symbol;Acc:HGNC:11735] | 6272 | -0.025 | -0.2649 | No |
| 87 | MARF1 | meiosis regulator and mRNA stability factor 1 [Source:HGNC Symbol;Acc:HGNC:29562] | 6304 | -0.027 | -0.2671 | No |
| 88 | CPEB1 | cytoplasmic polyadenylation element binding protein 1 [Source:HGNC Symbol;Acc:HGNC:21744] | 6435 | -0.038 | -0.2769 | No |
| 89 | RBM47 | RNA binding motif protein 47 [Source:HGNC Symbol;Acc:HGNC:30358] | 6443 | -0.038 | -0.2771 | No |
| 90 | SNIP1 | Smad nuclear interacting protein 1 [Source:HGNC Symbol;Acc:HGNC:30587] | 6466 | -0.040 | -0.2784 | No |
| 91 | DENR | density regulated re-initiation and release factor [Source:HGNC Symbol;Acc:HGNC:2769] | 6611 | -0.050 | -0.2892 | No |
| 92 | CPSF1 | cleavage and polyadenylation specific factor 1 [Source:HGNC Symbol;Acc:HGNC:2324] | 6629 | -0.052 | -0.2899 | No |
| 93 | RNF40 | ring finger protein 40 [Source:HGNC Symbol;Acc:HGNC:16867] | 6681 | -0.054 | -0.2934 | No |
| 94 | METTL3 | "methyltransferase 3, N6-adenosine-methyltransferase complex catalytic subunit [Source:HGNC Symbol;Acc:HGNC:17563]" | 6685 | -0.055 | -0.2930 | No |
| 95 | RBM15B | RNA binding motif protein 15B [Source:HGNC Symbol;Acc:HGNC:24303] | 6867 | -0.069 | -0.3065 | No |
| 96 | FUBP1 | far upstream element binding protein 1 [Source:HGNC Symbol;Acc:HGNC:4004] | 7018 | -0.081 | -0.3175 | No |
| 97 | RBMS1 | RNA binding motif single stranded interacting protein 1 [Source:HGNC Symbol;Acc:HGNC:9907] | 7121 | -0.090 | -0.3245 | No |
| 98 | CHTOP | chromatin target of PRMT1 [Source:HGNC Symbol;Acc:HGNC:24511] | 7184 | -0.095 | -0.3284 | No |
| 99 | PUM2 | pumilio RNA binding family member 2 [Source:HGNC Symbol;Acc:HGNC:14958] | 7186 | -0.095 | -0.3274 | No |
| 100 | CALR | calreticulin [Source:HGNC Symbol;Acc:HGNC:1455] | 7291 | -0.102 | -0.3345 | No |
| 101 | NOVA1 | NOVA alternative splicing regulator 1 [Source:HGNC Symbol;Acc:HGNC:7886] | 7394 | -0.110 | -0.3413 | No |
| 102 | ELAVL1 | ELAV like RNA binding protein 1 [Source:HGNC Symbol;Acc:HGNC:3312] | 7426 | -0.113 | -0.3425 | No |
| 103 | HNRNPC | heterogeneous nuclear ribonucleoprotein C [Source:HGNC Symbol;Acc:HGNC:5035] | 7437 | -0.114 | -0.3420 | No |
| 104 | DCP1A | decapping mRNA 1A [Source:HGNC Symbol;Acc:HGNC:18714] | 7525 | -0.121 | -0.3476 | No |
| 105 | RPL35 | ribosomal protein L35 [Source:HGNC Symbol;Acc:HGNC:10344] | 7563 | -0.124 | -0.3491 | No |
| 106 | EIF4A3 | eukaryotic translation initiation factor 4A3 [Source:HGNC Symbol;Acc:HGNC:18683] | 7646 | -0.130 | -0.3541 | No |
| 107 | MEX3D | mex-3 RNA binding family member D [Source:HGNC Symbol;Acc:HGNC:16734] | 7652 | -0.131 | -0.3531 | No |
| 108 | AQR | aquarius intron-binding spliceosomal factor [Source:HGNC Symbol;Acc:HGNC:29513] | 7654 | -0.131 | -0.3517 | No |
| 109 | CCT5 | chaperonin containing TCP1 subunit 5 [Source:HGNC Symbol;Acc:HGNC:1618] | 7690 | -0.134 | -0.3529 | No |
| 110 | TARDBP | TAR DNA binding protein [Source:HGNC Symbol;Acc:HGNC:11571] | 7864 | -0.146 | -0.3650 | No |
| 111 | CPSF6 | cleavage and polyadenylation specific factor 6 [Source:HGNC Symbol;Acc:HGNC:13871] | 7885 | -0.147 | -0.3649 | No |
| 112 | FMR1 | FMRP translational regulator 1 [Source:HGNC Symbol;Acc:HGNC:3775] | 7944 | -0.151 | -0.3678 | No |
| 113 | CPEB3 | cytoplasmic polyadenylation element binding protein 3 [Source:HGNC Symbol;Acc:HGNC:21746] | 8060 | -0.160 | -0.3751 | No |
| 114 | HNRNPM | heterogeneous nuclear ribonucleoprotein M [Source:HGNC Symbol;Acc:HGNC:5046] | 8127 | -0.165 | -0.3785 | No |
| 115 | RNPS1 | RNA binding protein with serine rich domain 1 [Source:HGNC Symbol;Acc:HGNC:10080] | 8275 | -0.177 | -0.3882 | No |
| 116 | RSL1D1 | ribosomal L1 domain containing 1 [Source:HGNC Symbol;Acc:HGNC:24534] | 8286 | -0.177 | -0.3870 | No |
| 117 | CSDC2 | cold shock domain containing C2 [Source:HGNC Symbol;Acc:HGNC:30359] | 8294 | -0.178 | -0.3855 | No |
| 118 | PCBP3 | poly(rC) binding protein 3 [Source:HGNC Symbol;Acc:HGNC:8651] | 8307 | -0.179 | -0.3845 | No |
| 119 | RBMS2 | RNA binding motif single stranded interacting protein 2 [Source:HGNC Symbol;Acc:HGNC:9909] | 8373 | -0.184 | -0.3876 | No |
| 120 | NCBP2 | nuclear cap binding protein subunit 2 [Source:HGNC Symbol;Acc:HGNC:7659] | 8478 | -0.192 | -0.3936 | No |
| 121 | RBPMS | "RNA binding protein, mRNA processing factor [Source:HGNC Symbol;Acc:HGNC:19097]" | 8547 | -0.197 | -0.3968 | No |
| 122 | THOC5 | THO complex 5 [Source:HGNC Symbol;Acc:HGNC:19074] | 8549 | -0.198 | -0.3947 | No |
| 123 | EXOSC8 | exosome component 8 [Source:HGNC Symbol;Acc:HGNC:17035] | 8554 | -0.198 | -0.3928 | No |
| 124 | HNRNPA2B1 | heterogeneous nuclear ribonucleoprotein A2/B1 [Source:HGNC Symbol;Acc:HGNC:5033] | 8576 | -0.200 | -0.3922 | No |
| 125 | HNRNPA3 | heterogeneous nuclear ribonucleoprotein A3 [Source:HGNC Symbol;Acc:HGNC:24941] | 8603 | -0.201 | -0.3920 | No |
| 126 | RBM8A | RNA binding motif protein 8A [Source:HGNC Symbol;Acc:HGNC:9905] | 8662 | -0.205 | -0.3943 | No |
| 127 | MECP2 | methyl-CpG binding protein 2 [Source:HGNC Symbol;Acc:HGNC:6990] | 8687 | -0.208 | -0.3939 | No |
| 128 | PABPC4 | poly(A) binding protein cytoplasmic 4 [Source:HGNC Symbol;Acc:HGNC:8557] | 8741 | -0.212 | -0.3957 | No |
| 129 | HNRNPL | heterogeneous nuclear ribonucleoprotein L [Source:HGNC Symbol;Acc:HGNC:5045] | 8892 | -0.224 | -0.4051 | No |
| 130 | RBMX | RNA binding motif protein X-linked [Source:HGNC Symbol;Acc:HGNC:9910] | 8938 | -0.227 | -0.4061 | No |
| 131 | RBMS3 | RNA binding motif single stranded interacting protein 3 [Source:HGNC Symbol;Acc:HGNC:13427] | 8981 | -0.230 | -0.4068 | No |
| 132 | UPF3B | UPF3B regulator of nonsense mediated mRNA decay [Source:HGNC Symbol;Acc:HGNC:20439] | 8986 | -0.230 | -0.4046 | No |
| 133 | RBMY1A1 | RNA binding motif protein Y-linked family 1 member A1 [Source:HGNC Symbol;Acc:HGNC:9912] | 9014 | -0.233 | -0.4041 | No |
| 134 | SAMD4A | sterile alpha motif domain containing 4A [Source:HGNC Symbol;Acc:HGNC:23023] | 9027 | -0.234 | -0.4024 | No |
| 135 | ZC3H12A | zinc finger CCCH-type containing 12A [Source:HGNC Symbol;Acc:HGNC:26259] | 9030 | -0.234 | -0.4000 | No |
| 136 | NCBP1 | nuclear cap binding protein subunit 1 [Source:HGNC Symbol;Acc:HGNC:7658] | 9149 | -0.244 | -0.4066 | No |
| 137 | SERBP1 | SERPINE1 mRNA binding protein 1 [Source:HGNC Symbol;Acc:HGNC:17860] | 9238 | -0.251 | -0.4107 | No |
| 138 | TRA2B | transformer 2 beta homolog [Source:HGNC Symbol;Acc:HGNC:10781] | 9243 | -0.251 | -0.4082 | No |
| 139 | ACO1 | aconitase 1 [Source:HGNC Symbol;Acc:HGNC:117] | 9426 | -0.266 | -0.4196 | No |
| 140 | LSM14A | LSM14A mRNA processing body assembly factor [Source:HGNC Symbol;Acc:HGNC:24489] | 9428 | -0.267 | -0.4167 | No |
| 141 | HNRNPR | heterogeneous nuclear ribonucleoprotein R [Source:HGNC Symbol;Acc:HGNC:5047] | 9430 | -0.267 | -0.4138 | No |
| 142 | ELAVL2 | ELAV like RNA binding protein 2 [Source:HGNC Symbol;Acc:HGNC:3313] | 9458 | -0.270 | -0.4130 | No |
| 143 | YTHDF3 | YTH N6-methyladenosine RNA binding protein 3 [Source:HGNC Symbol;Acc:HGNC:26465] | 9549 | -0.278 | -0.4170 | No |
| 144 | YBX1 | Y-box binding protein 1 [Source:HGNC Symbol;Acc:HGNC:8014] | 9623 | -0.285 | -0.4196 | No |
| 145 | RBM14 | RNA binding motif protein 14 [Source:HGNC Symbol;Acc:HGNC:14219] | 9663 | -0.288 | -0.4194 | No |
| 146 | SHMT1 | serine hydroxymethyltransferase 1 [Source:HGNC Symbol;Acc:HGNC:10850] | 9727 | -0.294 | -0.4211 | No |
| 147 | PCBP2 | poly(rC) binding protein 2 [Source:HGNC Symbol;Acc:HGNC:8648] | 9815 | -0.302 | -0.4246 | No |
| 148 | HNRNPU | heterogeneous nuclear ribonucleoprotein U [Source:HGNC Symbol;Acc:HGNC:5048] | 9847 | -0.306 | -0.4236 | No |
| 149 | RARA | retinoic acid receptor alpha [Source:HGNC Symbol;Acc:HGNC:9864] | 10141 | -0.335 | -0.4431 | Yes |
| 150 | PUM1 | pumilio RNA binding family member 1 [Source:HGNC Symbol;Acc:HGNC:14957] | 10145 | -0.335 | -0.4395 | Yes |
| 151 | RPL5 | ribosomal protein L5 [Source:HGNC Symbol;Acc:HGNC:10360] | 10201 | -0.341 | -0.4401 | Yes |
| 152 | ETF1 | eukaryotic translation termination factor 1 [Source:HGNC Symbol;Acc:HGNC:3477] | 10208 | -0.341 | -0.4367 | Yes |
| 153 | PTBP3 | polypyrimidine tract binding protein 3 [Source:HGNC Symbol;Acc:HGNC:10253] | 10259 | -0.347 | -0.4368 | Yes |
| 154 | RPL24 | ribosomal protein L24 [Source:HGNC Symbol;Acc:HGNC:10325] | 10263 | -0.347 | -0.4332 | Yes |
| 155 | SRSF1 | serine and arginine rich splicing factor 1 [Source:HGNC Symbol;Acc:HGNC:10780] | 10272 | -0.348 | -0.4299 | Yes |
| 156 | ELAVL3 | ELAV like RNA binding protein 3 [Source:HGNC Symbol;Acc:HGNC:3314] | 10273 | -0.348 | -0.4260 | Yes |
| 157 | SRSF5 | serine and arginine rich splicing factor 5 [Source:HGNC Symbol;Acc:HGNC:10787] | 10282 | -0.349 | -0.4228 | Yes |
| 158 | ADARB1 | adenosine deaminase RNA specific B1 [Source:HGNC Symbol;Acc:HGNC:226] | 10295 | -0.350 | -0.4198 | Yes |
| 159 | LUC7L2 | "LUC7 like 2, pre-mRNA splicing factor [Source:HGNC Symbol;Acc:HGNC:21608]" | 10334 | -0.354 | -0.4188 | Yes |
| 160 | ARC | activity regulated cytoskeleton associated protein [Source:HGNC Symbol;Acc:HGNC:648] | 10364 | -0.357 | -0.4171 | Yes |
| 161 | ELAVL4 | ELAV like RNA binding protein 4 [Source:HGNC Symbol;Acc:HGNC:3315] | 10412 | -0.362 | -0.4168 | Yes |
| 162 | TEX13B | testis expressed 13B [Source:HGNC Symbol;Acc:HGNC:11736] | 10427 | -0.364 | -0.4138 | Yes |
| 163 | CSTF2 | cleavage stimulation factor subunit 2 [Source:HGNC Symbol;Acc:HGNC:2484] | 10438 | -0.366 | -0.4106 | Yes |
| 164 | RBM15 | RNA binding motif protein 15 [Source:HGNC Symbol;Acc:HGNC:14959] | 10499 | -0.373 | -0.4111 | Yes |
| 165 | ZFP36L2 | ZFP36 ring finger protein like 2 [Source:HGNC Symbol;Acc:HGNC:1108] | 10509 | -0.374 | -0.4077 | Yes |
| 166 | PCF11 | PCF11 cleavage and polyadenylation factor subunit [Source:HGNC Symbol;Acc:HGNC:30097] | 10521 | -0.376 | -0.4043 | Yes |
| 167 | RPS14 | ribosomal protein S14 [Source:HGNC Symbol;Acc:HGNC:10387] | 10558 | -0.379 | -0.4029 | Yes |
| 168 | EXOSC9 | exosome component 9 [Source:HGNC Symbol;Acc:HGNC:9137] | 10606 | -0.384 | -0.4024 | Yes |
| 169 | RPS7 | ribosomal protein S7 [Source:HGNC Symbol;Acc:HGNC:10440] | 10608 | -0.384 | -0.3981 | Yes |
| 170 | DHX9 | DExH-box helicase 9 [Source:HGNC Symbol;Acc:HGNC:2750] | 10627 | -0.386 | -0.3953 | Yes |
| 171 | HNRNPAB | heterogeneous nuclear ribonucleoprotein A/B [Source:HGNC Symbol;Acc:HGNC:5034] | 10632 | -0.386 | -0.3913 | Yes |
| 172 | LUC7L3 | LUC7 like 3 pre-mRNA splicing factor [Source:HGNC Symbol;Acc:HGNC:24309] | 10647 | -0.388 | -0.3880 | Yes |
| 173 | SRSF6 | serine and arginine rich splicing factor 6 [Source:HGNC Symbol;Acc:HGNC:10788] | 10659 | -0.389 | -0.3846 | Yes |
| 174 | YTHDC1 | YTH domain containing 1 [Source:HGNC Symbol;Acc:HGNC:30626] | 10687 | -0.391 | -0.3823 | Yes |
| 175 | TUT1 | "terminal uridylyl transferase 1, U6 snRNA-specific [Source:HGNC Symbol;Acc:HGNC:26184]" | 10699 | -0.393 | -0.3788 | Yes |
| 176 | CNBP | CCHC-type zinc finger nucleic acid binding protein [Source:HGNC Symbol;Acc:HGNC:13164] | 10707 | -0.394 | -0.3750 | Yes |
| 177 | RBM25 | RNA binding motif protein 25 [Source:HGNC Symbol;Acc:HGNC:23244] | 10800 | -0.405 | -0.3777 | Yes |
| 178 | CELF2 | CUGBP Elav-like family member 2 [Source:HGNC Symbol;Acc:HGNC:2550] | 10832 | -0.409 | -0.3756 | Yes |
| 179 | RBM5 | RNA binding motif protein 5 [Source:HGNC Symbol;Acc:HGNC:9902] | 10849 | -0.410 | -0.3723 | Yes |
| 180 | TIA1 | TIA1 cytotoxic granule associated RNA binding protein [Source:HGNC Symbol;Acc:HGNC:11802] | 10855 | -0.411 | -0.3681 | Yes |
| 181 | NUP98 | nucleoporin 98 and 96 precursor [Source:HGNC Symbol;Acc:HGNC:8068] | 10875 | -0.413 | -0.3649 | Yes |
| 182 | GNL3 | G protein nucleolar 3 [Source:HGNC Symbol;Acc:HGNC:29931] | 10907 | -0.418 | -0.3627 | Yes |
| 183 | SF3B1 | splicing factor 3b subunit 1 [Source:HGNC Symbol;Acc:HGNC:10768] | 10948 | -0.424 | -0.3611 | Yes |
| 184 | LARP1 | "La ribonucleoprotein 1, translational regulator [Source:HGNC Symbol;Acc:HGNC:29531]" | 10964 | -0.425 | -0.3576 | Yes |
| 185 | TIAL1 | TIA1 cytotoxic granule associated RNA binding protein like 1 [Source:HGNC Symbol;Acc:HGNC:11804] | 11049 | -0.438 | -0.3593 | Yes |
| 186 | RC3H2 | ring finger and CCCH-type domains 2 [Source:HGNC Symbol;Acc:HGNC:21461] | 11102 | -0.444 | -0.3585 | Yes |
| 187 | G3BP1 | G3BP stress granule assembly factor 1 [Source:HGNC Symbol;Acc:HGNC:30292] | 11117 | -0.445 | -0.3546 | Yes |
| 188 | SYNCRIP | synaptotagmin binding cytoplasmic RNA interacting protein [Source:HGNC Symbol;Acc:HGNC:16918] | 11182 | -0.454 | -0.3546 | Yes |
| 189 | RPS5 | ribosomal protein S5 [Source:HGNC Symbol;Acc:HGNC:10426] | 11206 | -0.458 | -0.3513 | Yes |
| 190 | PIWIL2 | piwi like RNA-mediated gene silencing 2 [Source:HGNC Symbol;Acc:HGNC:17644] | 11219 | -0.460 | -0.3471 | Yes |
| 191 | RPS3 | ribosomal protein S3 [Source:HGNC Symbol;Acc:HGNC:10420] | 11245 | -0.463 | -0.3439 | Yes |
| 192 | AGO2 | argonaute RISC catalytic component 2 [Source:HGNC Symbol;Acc:HGNC:3263] | 11246 | -0.463 | -0.3387 | Yes |
| 193 | KHSRP | KH-type splicing regulatory protein [Source:HGNC Symbol;Acc:HGNC:6316] | 11285 | -0.469 | -0.3365 | Yes |
| 194 | EIF4G2 | eukaryotic translation initiation factor 4 gamma 2 [Source:HGNC Symbol;Acc:HGNC:3297] | 11313 | -0.473 | -0.3333 | Yes |
| 195 | NCBP3 | nuclear cap binding subunit 3 [Source:HGNC Symbol;Acc:HGNC:24612] | 11502 | -0.501 | -0.3426 | Yes |
| 196 | CELF3 | CUGBP Elav-like family member 3 [Source:HGNC Symbol;Acc:HGNC:11967] | 11510 | -0.502 | -0.3375 | Yes |
| 197 | NOCT | nocturnin [Source:HGNC Symbol;Acc:HGNC:14254] | 11566 | -0.510 | -0.3362 | Yes |
| 198 | LUC7L | LUC7 like [Source:HGNC Symbol;Acc:HGNC:6723] | 11627 | -0.521 | -0.3351 | Yes |
| 199 | CASC3 | CASC3 exon junction complex subunit [Source:HGNC Symbol;Acc:HGNC:17040] | 11630 | -0.522 | -0.3294 | Yes |
| 200 | SF1 | splicing factor 1 [Source:HGNC Symbol;Acc:HGNC:12950] | 11664 | -0.527 | -0.3262 | Yes |
| 201 | SECISBP2L | SECIS binding protein 2 like [Source:HGNC Symbol;Acc:HGNC:28997] | 11688 | -0.530 | -0.3221 | Yes |
| 202 | LARP4B | La ribonucleoprotein 4B [Source:HGNC Symbol;Acc:HGNC:28987] | 11695 | -0.531 | -0.3166 | Yes |
| 203 | LARP4 | La ribonucleoprotein 4 [Source:HGNC Symbol;Acc:HGNC:24320] | 11700 | -0.533 | -0.3110 | Yes |
| 204 | SUPT5H | "SPT5 homolog, DSIF elongation factor subunit [Source:HGNC Symbol;Acc:HGNC:11469]" | 11735 | -0.539 | -0.3076 | Yes |
| 205 | PUM3 | pumilio RNA binding family member 3 [Source:HGNC Symbol;Acc:HGNC:29676] | 11771 | -0.545 | -0.3043 | Yes |
| 206 | ZC3H11A | zinc finger CCCH-type containing 11A [Source:HGNC Symbol;Acc:HGNC:29093] | 11780 | -0.546 | -0.2988 | Yes |
| 207 | JRK | Jrk helix-turn-helix protein [Source:HGNC Symbol;Acc:HGNC:6199] | 11830 | -0.555 | -0.2965 | Yes |
| 208 | SSB | small RNA binding exonuclease protection factor La [Source:HGNC Symbol;Acc:HGNC:11316] | 11955 | -0.584 | -0.2998 | Yes |
| 209 | NOVA2 | NOVA alternative splicing regulator 2 [Source:HGNC Symbol;Acc:HGNC:7887] | 12006 | -0.594 | -0.2971 | Yes |
| 210 | YBX3 | Y-box binding protein 3 [Source:HGNC Symbol;Acc:HGNC:2428] | 12024 | -0.599 | -0.2917 | Yes |
| 211 | RBM38 | RNA binding motif protein 38 [Source:HGNC Symbol;Acc:HGNC:15818] | 12026 | -0.599 | -0.2851 | Yes |
| 212 | SNRNP70 | small nuclear ribonucleoprotein U1 subunit 70 [Source:HGNC Symbol;Acc:HGNC:11150] | 12073 | -0.613 | -0.2819 | Yes |
| 213 | SPEN | spen family transcriptional repressor [Source:HGNC Symbol;Acc:HGNC:17575] | 12108 | -0.622 | -0.2776 | Yes |
| 214 | EIF4A1 | eukaryotic translation initiation factor 4A1 [Source:HGNC Symbol;Acc:HGNC:3282] | 12111 | -0.623 | -0.2708 | Yes |
| 215 | EIF4G1 | eukaryotic translation initiation factor 4 gamma 1 [Source:HGNC Symbol;Acc:HGNC:3296] | 12122 | -0.625 | -0.2647 | Yes |
| 216 | RPL13A | ribosomal protein L13a [Source:HGNC Symbol;Acc:HGNC:10304] | 12200 | -0.645 | -0.2635 | Yes |
| 217 | PTBP2 | polypyrimidine tract binding protein 2 [Source:HGNC Symbol;Acc:HGNC:17662] | 12211 | -0.649 | -0.2571 | Yes |
| 218 | FXR2 | FMR1 autosomal homolog 2 [Source:HGNC Symbol;Acc:HGNC:4024] | 12219 | -0.650 | -0.2504 | Yes |
| 219 | IGF2BP2 | insulin like growth factor 2 mRNA binding protein 2 [Source:HGNC Symbol;Acc:HGNC:28867] | 12244 | -0.657 | -0.2449 | Yes |
| 220 | SAMD4B | sterile alpha motif domain containing 4B [Source:HGNC Symbol;Acc:HGNC:25492] | 12247 | -0.659 | -0.2377 | Yes |
| 221 | CPSF7 | cleavage and polyadenylation specific factor 7 [Source:HGNC Symbol;Acc:HGNC:30098] | 12251 | -0.661 | -0.2306 | Yes |
| 222 | NCL | nucleolin [Source:HGNC Symbol;Acc:HGNC:7667] | 12255 | -0.662 | -0.2234 | Yes |
| 223 | PABPC3 | poly(A) binding protein cytoplasmic 3 [Source:HGNC Symbol;Acc:HGNC:8556] | 12257 | -0.663 | -0.2161 | Yes |
| 224 | DND1 | DND microRNA-mediated repression inhibitor 1 [Source:HGNC Symbol;Acc:HGNC:23799] | 12266 | -0.667 | -0.2092 | Yes |
| 225 | RPS13 | ribosomal protein S13 [Source:HGNC Symbol;Acc:HGNC:10386] | 12334 | -0.687 | -0.2069 | Yes |
| 226 | QKI | "QKI, KH domain containing RNA binding [Source:HGNC Symbol;Acc:HGNC:21100]" | 12340 | -0.688 | -0.1996 | Yes |
| 227 | HNRNPK | heterogeneous nuclear ribonucleoprotein K [Source:HGNC Symbol;Acc:HGNC:5044] | 12341 | -0.688 | -0.1919 | Yes |
| 228 | ILF3 | interleukin enhancer binding factor 3 [Source:HGNC Symbol;Acc:HGNC:6038] | 12353 | -0.692 | -0.1850 | Yes |
| 229 | LSM14B | LSM family member 14B [Source:HGNC Symbol;Acc:HGNC:15887] | 12361 | -0.695 | -0.1778 | Yes |
| 230 | CELF1 | CUGBP Elav-like family member 1 [Source:HGNC Symbol;Acc:HGNC:2549] | 12391 | -0.704 | -0.1722 | Yes |
| 231 | EIF3A | eukaryotic translation initiation factor 3 subunit A [Source:HGNC Symbol;Acc:HGNC:3271] | 12402 | -0.707 | -0.1651 | Yes |
| 232 | NXF1 | nuclear RNA export factor 1 [Source:HGNC Symbol;Acc:HGNC:8071] | 12410 | -0.710 | -0.1577 | Yes |
| 233 | SECISBP2 | SECIS binding protein 2 [Source:HGNC Symbol;Acc:HGNC:30972] | 12416 | -0.712 | -0.1502 | Yes |
| 234 | RPL7 | ribosomal protein L7 [Source:HGNC Symbol;Acc:HGNC:10363] | 12519 | -0.757 | -0.1498 | Yes |
| 235 | RPS2 | ribosomal protein S2 [Source:HGNC Symbol;Acc:HGNC:10404] | 12541 | -0.769 | -0.1428 | Yes |
| 236 | PABPC1 | poly(A) binding protein cytoplasmic 1 [Source:HGNC Symbol;Acc:HGNC:8554] | 12562 | -0.778 | -0.1357 | Yes |
| 237 | SRRM2 | serine/arginine repetitive matrix 2 [Source:HGNC Symbol;Acc:HGNC:16639] | 12666 | -0.837 | -0.1345 | Yes |
| 238 | RPL41 | ribosomal protein L41 [Source:HGNC Symbol;Acc:HGNC:10354] | 12674 | -0.847 | -0.1256 | Yes |
| 239 | DAZAP1 | DAZ associated protein 1 [Source:HGNC Symbol;Acc:HGNC:2683] | 12686 | -0.854 | -0.1169 | Yes |
| 240 | THOC2 | THO complex 2 [Source:HGNC Symbol;Acc:HGNC:19073] | 12691 | -0.855 | -0.1077 | Yes |
| 241 | YTHDF2 | YTH N6-methyladenosine RNA binding protein 2 [Source:HGNC Symbol;Acc:HGNC:31675] | 12694 | -0.860 | -0.0982 | Yes |
| 242 | FUS | FUS RNA binding protein [Source:HGNC Symbol;Acc:HGNC:4010] | 12739 | -0.902 | -0.0916 | Yes |
| 243 | SRSF4 | serine and arginine rich splicing factor 4 [Source:HGNC Symbol;Acc:HGNC:10786] | 12749 | -0.911 | -0.0822 | Yes |
| 244 | RPS3A | ribosomal protein S3A [Source:HGNC Symbol;Acc:HGNC:10421] | 12758 | -0.921 | -0.0725 | Yes |
| 245 | DDX3X | DEAD-box helicase 3 X-linked [Source:HGNC Symbol;Acc:HGNC:2745] | 12788 | -0.949 | -0.0642 | Yes |
| 246 | CLUH | clustered mitochondria homolog [Source:HGNC Symbol;Acc:HGNC:29094] | 12824 | -1.013 | -0.0556 | Yes |
| 247 | TAF15 | TATA-box binding protein associated factor 15 [Source:HGNC Symbol;Acc:HGNC:11547] | 12828 | -1.023 | -0.0444 | Yes |
| 248 | DDX5 | DEAD-box helicase 5 [Source:HGNC Symbol;Acc:HGNC:2746] | 12834 | -1.038 | -0.0332 | Yes |
| 249 | ZFP36 | ZFP36 ring finger protein [Source:HGNC Symbol;Acc:HGNC:12862] | 12846 | -1.052 | -0.0223 | Yes |
| 250 | EDC3 | enhancer of mRNA decapping 3 [Source:HGNC Symbol;Acc:HGNC:26114] | 12868 | -1.123 | -0.0114 | Yes |
| 251 | ZFP36L1 | ZFP36 ring finger protein like 1 [Source:HGNC Symbol;Acc:HGNC:1107] | 12888 | -1.244 | 0.0009 | Yes |
Table: GSEA details [plain text format]

  

Fig 2: GOMF\_MRNA\_BINDING      
 Blue-Pink O' Gram in the Space of the Analyzed GeneSet

  

Fig 3: GOMF\_MRNA\_BINDING: Random ES distribution      
 Gene set null distribution of ES for **GOMF\_MRNA\_BINDING**
